# Asymmetric integration of various cancer datasets for identifying risk-associated variants and genes

**DOI:** 10.1093/bioadv/vbaf253

**Published:** 2025-10-14

**Authors:** Ruixuan Wang, Lam Tran, Benjamin Brennan, Lars G Fritsche, Kevin He, J Chad Brenner, Hui Jiang

**Affiliations:** Department of Biostatistics, School of Public Health, University of Michigan, Ann Arbor, MI 48109, United States; Department of Biostatistics, School of Public Health, University of Michigan, Ann Arbor, MI 48109, United States; Department of Biostatistics, School of Public Health, University of Michigan, Ann Arbor, MI 48109, United States; Department of Biostatistics, School of Public Health, University of Michigan, Ann Arbor, MI 48109, United States; Center for Statistical Genetics, University of Michigan, Ann Arbor, MI 48109, United States; Department of Biostatistics, School of Public Health, University of Michigan, Ann Arbor, MI 48109, United States; Department of Otolaryngology-Head and Neck Surgery, University of Michigan, Ann Arbor, MI 48109, United States; Department of Biostatistics, School of Public Health, University of Michigan, Ann Arbor, MI 48109, United States; Center for Statistical Genetics, University of Michigan, Ann Arbor, MI 48109, United States

## Abstract

**Motivation:**

Cancer genomic research provides an opportunity to identify cancer risk-associated genes, but often suffers from undesirable low statistical power due to a limited sample size. Integrated analysis with different cancers has the potential to enhance statistical power for identifying pan-cancer risk genes. However, substantial heterogeneity across various cancers makes this challenging.

**Results:**

Recently, a novel asymmetric integration method was developed that can deal with data heterogeneity and exclude unhelpful datasets from the analysis. We adapted and applied this method to integrate genotype datasets with matched case and control individuals from the Michigan Genomics Initiative, using each cancer as the primary dataset of interest and the other cancers as auxiliary datasets, respectively. Conditional logistic regression models were coupled with the asymmetric integrated framework to handle the matched case–control study design and permutation tests were performed to control for false discovery rates (FDRs). At the same FDR level, the integrated analysis found more potential genetic variants and genes that are associated with the risks of various cancers, showcasing the promise of the proposed approach for integrated analysis of cancer datasets.

**Availability and implementation:**

Our method is available as source code at https://github.com/rxxwang/integrate_cancer.

## 1 Introduction

Cancer is one of the leading causes of death in the USA and the world (Twombly 2005). Aside from environmental and aging factors, hereditary components raise more and more attention in scientific research. Genomic datasets and research on cancers help improve diagnosis and treatment plans customized to individual patients, a paradigm known as precision medicine (Nakagawa and Fujita 2018). In recent years, individualized precision medicine approaches have shown promise in bringing effective therapeutics to cancer patients (Gazdar and Minna 2013). Since each cancer patient has a unique genetic profile, customizing therapeutic options for the individual will benefit more patients, instead of the conventional “one-size-fits-all” approach in cancer treatment (Shin *et al.* 2017). Using genomic techniques, one can measure the expression or test genetic variants in thousands of genes simultaneously which allows the monitoring of molecular variation on a genome-wide scale (Schena *et al.* 1995). When coupled with tailored statistical and computational methods, these genomic data allow one to identify cancer-related genes that can lead to more accurate risk prediction, more precise diagnosis, more reliable prognosis, and better treatment regimens (Quackenbush 2006). The data-driven nature of the precision medicine paradigm relies heavily on effective strategies in data generation, collection, and analysis. It also relies critically on the availability of large-scale data. In recent years, genomic datasets from thousands of patients with a wide range of cancer types have been generated from many cancer studies (Tomczak *et al.* 2015). These datasets provide unprecedented information for the study of biological mechanisms underlying cancer etiology as well as for the identification of clinically relevant and effective biomarkers with high discriminative and predictive powers which are essential for the success of precision medicine.

While cancer genomic datasets have contributed tremendously to cancer-related gene and biomarker discovery, due to the nature of some cancers being relatively rare diseases, existing cancer genomic datasets often are of relatively small sample sizes, i.e. in hundreds or low thousands, especially for rare cancer types. The scarcity of data severely limits the statistical power for finding biologically and clinically relevant genetic variants and genes. As a result, inference tends to be unstable, misleading, or even invalid due to high statistical uncertainty. Such limitations may have partly contributed to the lack of reproducibility in cancer research (Bensalah *et al.* 2007, Hatzis *et al.* 2014, Liang *et al.* 2019). In the pursuit of more reliable and accurate methods of identification, it was recognized that different cancer types may share common underlying genetic pathways, and pan-cancer analysis has the potential to identify such common genes by taking advantage of the increased sample size when integrating datasets from different cancer types (Weinstein *et al.* 2013, Freeman *et al.* 2020, ICGC/TCGA Pan-Cancer Analysis of Whole Genomes Consortium 2020). The challenge for such integrated analysis lies in the fact that it requires the accommodation of the substantial heterogeneity across different cancer types, or different subtypes of a single cancer. Simply pooling all the datasets together (ICGC/TCGA Pan-Cancer Analysis of Whole Genomes Consortium 2020) or naively looking for overlapped findings or correlations (Rashkin *et al.* 2020) across different cancer types will dilute the signal and may lead to lower statistical power and/or more false discoveries, partly due to irrelevant cancer types or datasets of unknown quality or confounding factors being included in the analysis without careful selection, screening, and cleaning. There is a lack of tailored and sophisticated statistical and computational methods for such integrated data analysis to facilitate more powerful discovery while reducing false discovery and consequently improving reproducibility.

For each dataset on one specific primary cancer of interest, we aim to integrate additional auxiliary datasets from other cancers to increase the sample size and enhance statistical power. Concurrently, we seek to avoid the spurious effects of irrelevant signals from the auxiliary datasets, which could compromise the validity of our scientific conclusions. Recently, a novel asymmetric data integration method has been developed to solve this problem and address the potential heterogeneity present in the datasets (Tran *et al.* 2023). This method assigns data-adaptive weights to the auxiliary datasets determined by minimizing the leave-one-out cross-validation (LOOCV) metric in the primary dataset. As a result, a lower weight will reduce the relevance of an auxiliary dataset and a zero weight will completely exclude an unhelpful dataset from the analysis. Here, we adapt, extend, and apply this method to integrate data from various cancers to identify potential genetic variants and genes that are associated with cancer risk.

Our data were extracted from the Michigan Genomics Initiative (MGI) (Zawistowski *et al.* 2023), which represents an ongoing research endeavor at the University of Michigan integrating electronic health records with corresponding genetic data from Michigan Medicine patients and aiming to derive novel biomedical insights. Given that some cancers are relatively rare diseases, case–control studies are an effective approach to assess the exposures and factors that increase cancer risk. We compiled case–control genotype datasets with associated clinical information for 14 different cancer types (Table 1), with each cancer patient matched to a non-cancer control by gender, race (self-reported), age (current age or age at death), and ZIP code. While the original asymmetric data integration method in Tran *et al.* (2023) was developed to work with linear and Cox regression models, here we couple conditional logistic regression models with the asymmetric data integration framework to infer cancer risk-associated germline variants and genes in our matched case–control study, as they effectively control for variables that may confound environmental factors and improve estimation efficiency (Chen and Rodriguez 2007). Besides integrated data analysis, we also applied conditional logistic regression on each primary cancer dataset of interest individually to compare its performance with the integrated method. As expected, we found that incorporating auxiliary cancer datasets can enhance the statistical power for the discovery of potential cancer risk-associated germline variants and genes. In the future, the identified variants and genes can potentially be used in predictive modeling to advance scientific research and clinical applications.

**Table 1.** Cancer types and stratum sample sizes.

Cancer type	# Strata	Cancer type	# Strata
Bladder	554	Liver	260
Brain	334	Lung	394
Breast	1964	Ovarian	158
Esophageal	168	Pancreatic	233
Head and neck	670	Prostate	1079
Kidney	680	Sarcoma	654
Leukemia	71	Stomach	76

The remainder of this article is organized as follows: Section 2 briefly introduces the asymmetric integration framework and our implementation of the conditional logistic regression models. We will also introduce the MGI dataset, the method for the integrated association analysis, as well as the approach for controlling the false discovery rates (FDRs). Section 3 presents the results of the integrated association analysis, followed by a discussion in Section 4.

## 2 Methods

**Figure 1.**
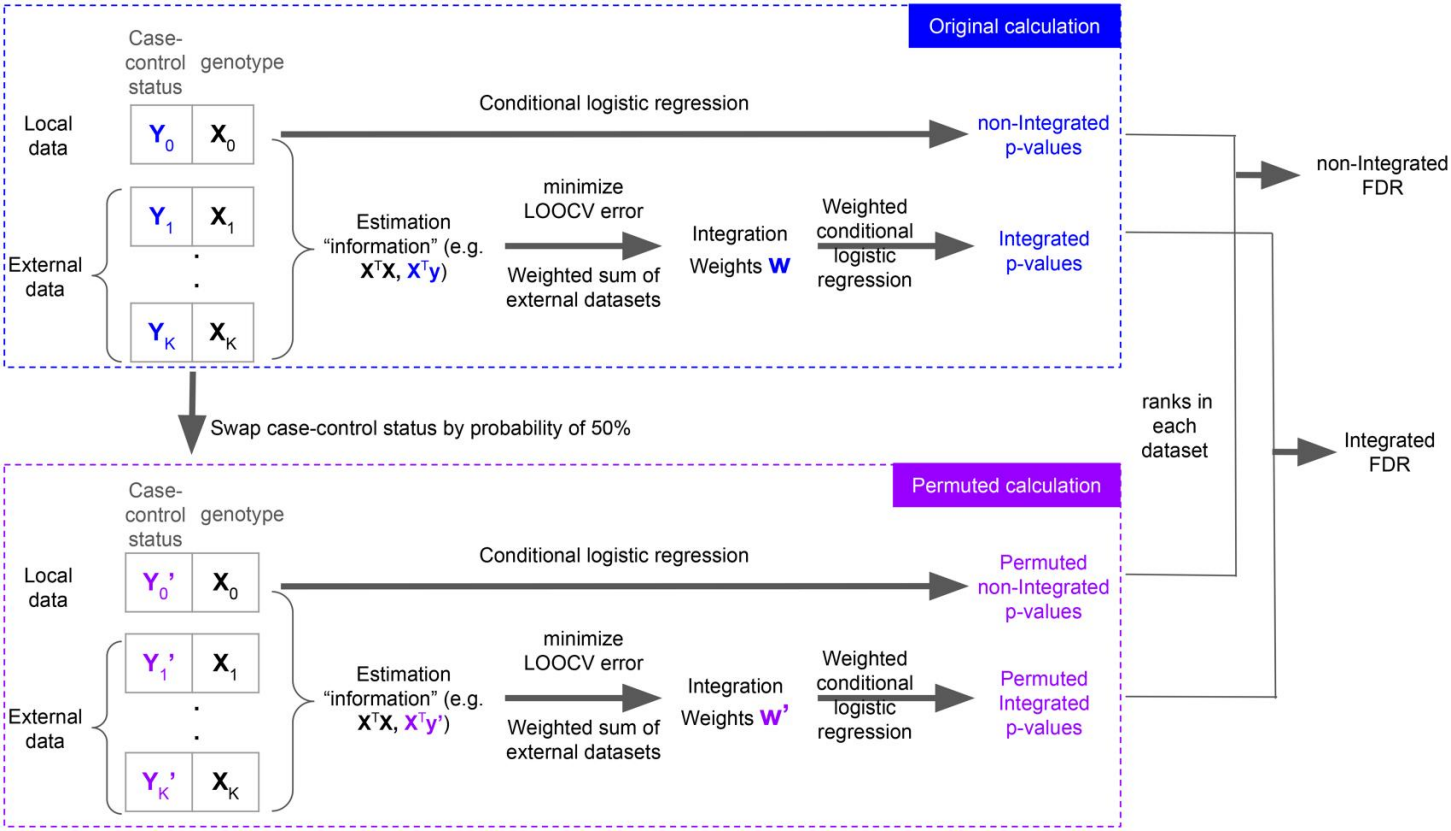
Flowchart of the methodology used in this article. We suppose that the local dataset has predictor (genotype) $X_{0}$ and respond (case–control status) $y_{0}$, and the K external datasets have predictor $X_{1}$, …, $X_{K}$ and respond $y_{1}$, …, $y_{K}$.

Figure 1 presents a flowchart illustrating the methodology used in this article. With the genotype and case–control status from the MGI cohort (Section 2.3), we first applied conditional logistic regression to the dataset and then utilized the asymmetric data integration method (Section 2.1) to weighted conditional logistic regression (Section 2.2). To compare the *P*-values generated from these two methods, we permuted the case–control status with a probability of 50% and implemented a single-iteration permutation method to calculate the FDR (Section 2.4).

### 2.1 Asymmetric data integration

In Tran *et al.* (2023), Tran *et al.* proposed a novel asymmetric data integration method based on a weighted regression framework. Here, we briefly review it and follow its terminology and notations. Suppose the local dataset (i.e. the dataset for the primary cancer of interest) has ${\hat{\beta }}_{0}$ as the estimated parameter vector and there are also *K* candidate external datasets (i.e. the auxiliary datasets from other cancers), with ${\hat{\beta }}_{i}$ the parameter vector estimated from the *i*th external dataset, i=1,…,K. Following the asymmetric data integration framework, data-adaptive integration weights ω will be assigned to the *K* external datasets by solving an optimization problem. Define the sample size of the local dataset as $n_{0}$. Let ${\hat{\beta }}_{0}^{⋆}$ be the estimates for the parameters of the local dataset after integration and $\ell_{-j}$ be leave-one-out likelihood constructed by the local dataset removing the *j*th sample, $j=1,\ldots ,n_{0}$. Correspondingly, ${\hat{\beta }}_{0-j}^{⋆}$ is the leave-one-out parameter estimates which maximize $\ell_{-j}$. The objective function to be minimized is the negative LOOCV log-likelihood in the local dataset:


(1) $$\begin{array}{c} \underset{\omega }{\arg\min}\sum_{j=1}^{n_{0}}\ell_{-j}({\hat{\beta }}_{0-j}^{⋆})-\ell ({\hat{\beta }}_{0-j}^{⋆})\\ \text{subject}\;\text{to}\;\omega_{i}\in \left[0,1\right]\;\text{for}\;i\in 1,\ldots ,K \end{array}$$

To compute ${\hat{\beta }}_{0}^{⋆}$, the Newton–Raphson (N-R) algorithm is used to maximize the log-likelihood, with the score vector and Hessian matrix in each NR update constructed as weighted sums. To accelerate the computation, a second-order Taylor expansion of the derivative of $\ell_{\left(-j\right)}$ around ${\hat{\beta }}_{0-j}^{⋆}$ yields the following simplified expression:


$${\hat{\beta }}_{0-j}^{⋆}\approx {\hat{\beta }}_{0}^{⋆}-{\left\{\frac{\partial^{2}\ell_{-j}({\hat{\beta }}_{0}^{⋆})}{\partial \beta \partial \beta^{T}}\right\}}^{-1}\frac{\partial \ell_{-j}({\hat{\beta }}_{0}^{⋆})}{\partial \beta }$$

To further accelerate the computation, a reduced space optimization algorithm was proposed in order to minimize the LOOCV error over only two parameters. We will sketch the procedure in the following section and more details can be found in Tran *et al.* (2023).

### 2.2 Asymmetric data integration with conditional logistic regression

The original asymmetric data integration method (Tran *et al.* 2023) was developed to work with linear and Cox regression models. In this article, we adapt and extend this framework to couple it with conditional logistic regression. Conditional logistic regression is a generalized linear regression model that extends logistic regression by incorporating stratification and matching. Consider matched case–control data where each stratum *i* is ordered as case first and control second (i.e. $y_{i1}=1$ and $y_{i2}=0$). Suppose there are *n* subjects (i.e. n/2 case–control pairs or strata). The data matrix and the parameter vector are $X\in R^{n\times p}$ and $\beta \in R^{p\times 1}$, respectively. The conditional logistic log-likelihood is given by


$$\begin{array}{c} \ell (\beta )=\sum_{i}\left(X_{i1}-X_{i2}\right)\beta -\text{log}\;\left(1+\text{exp}\;\left(\left(X_{i1}-X_{i2}\right)\beta \right)\right)\\ =\sum_{i}Z_{i}\beta -\text{log}\;\left(1+\text{exp}\;\left(Z_{i}\beta \right)\right) \end{array}$$

where $Z_{i}=X_{i1}-X_{i2}$. The matrix $Z\in R^{n/2\times p}$ collects this difference for all strata.

The corresponding gradient and Hessian matrix are crucial in the process of maximizing the log-likelihood using the Newton–Raphson algorithm. Consequently, we first derived these two components of the conditional logistic model. The corresponding gradient, which represents the first-order partial derivatives of the log-likelihood, is given by


$$\begin{array}{c} g(\beta )=\frac{\partial \ell }{\partial \beta }=\sum_{i}Z_{i}^{T}-\frac{Z_{i}^{T}\;\text{exp}\;\left(X_{i1}\beta \right)}{\;\text{exp}\;\left(X_{i1}\beta \right)+\text{exp}\;\left(X_{i2}\beta \right)}\\ =Z^{T}\left(y^{⋆}-\mu \right) \end{array}$$

where $y^{⋆}$ is a length n/2 vector with elements $y_{i}^{⋆}=y_{i1}-y_{i2}=1$ and μ is also a length n/2 vector with elements $(\text{exp}\;\left(X_{i1}\beta \right)/\left(\text{exp}\;\left(X_{i1}\beta \right)+\text{exp}\;\left(X_{i2}\beta \right)\right)$.

The Hessian matrix, which is a square matrix of second-order partial derivatives of log-likelihood, is given by


$$\begin{array}{c} H(\beta )=\frac{\partial^{2}\ell }{\partial \beta^{2}}=\sum_{i}-\frac{Z_{i}^{T}\;\text{exp}\;\left(\left(X_{i1}+X_{i2}\right)\beta \right)Z_{i}}{{\left(\text{exp}\;\left(X_{i1}\beta \right)+\text{exp}\;\left(X_{i2}\beta \right)\right)}^{2}}\\ =-Z^{T}\mathrm{WZ} \end{array}$$

where W is a n/2 × n/2 matrix with diagonal elements $\text{exp}\;\left(\left(X_{i1}+X_{i2}\right)\beta \right)/{\left(\text{exp}\;\left(X_{i1}\beta \right)+\text{exp}\;\left(X_{i2}\beta \right)\right)}^{2}$ and non-diagonal elements 0.

Similar to the Cox model in Tran *et al.* (2023), the objective function to be minimized here is the negative cross-validated log-likelihood. The leave-one-out estimate of conditional logistic regression for each stratum in the local data left out is as follows:


$$\begin{array}{cc} {\hat{\beta }}_{0-j}^{⋆}\approx {\hat{\beta }}_{0}^{⋆}-{\left\{\frac{\partial^{2}\ell_{-j}({\hat{\beta }}_{0}^{⋆})}{\partial \beta \partial \beta^{T}}\right\}}^{-1}\frac{\partial \ell_{-j}({\hat{\beta }}_{0}^{⋆})}{\partial \beta } & \\ ={\hat{\beta }}_{0}^{⋆}-\frac{{\left(Z_{0}^{T}W_{0}Z_{0}+\sum_{k=1}^{K}\omega_{k}Z_{k}^{T}W_{k}Z_{k}\right)}^{-1}Z_{i}^{T}\left(1-\mu_{i}\right)}{1-v_{ii}} & \end{array}$$

where $v_{ii}$ is the *i*th diagonal of the matrix $V=W_{0}^{1/2}Z_{0}{\left(Z_{0}^{T}W_{0}Z_{0}+\sum_{k=1}^{K}\omega_{k}Z_{k}^{T}W_{k}Z_{k}\right)}^{-1}Z_{0}^{T}W_{0}^{1/2}$. This is equivalent to taking one step of a Newton–Raphson algorithm in the direction of the left-out strata, weighted by its contribution to the matrix V.

Finally, these leave-one-out estimates are used to maximize the cross-validated log-likelihood, equivalent to the summing over the likelihood of each stratum using an estimate leaving out that stratum as follows:


$$\sum_{i}\ell ({\hat{\beta }}_{-i})-\ell_{-i}({\hat{\beta }}_{-i})=\sum_{i}\ell_{i}({\hat{\beta }}_{-i})$$

To optimize the LOOCV log-likelihood in Equation (1), we need to solve a high-dimensional optimization problem, with one weight parameter $w_{i}$ for each external dataset. To reduce the computational burden, here we use the technique in Tran *et al.* (2023) to reduce it to a two-dimensional optimization problem. We first apply the log-likelihood ratio test for each external data to generate a *P*-value $q_{i}$. Then we form a mapping $q_{i}\;\mapsto \;\omega_{i}$ with two parameters $q_{A}$ and $q_{B}$ as follows: $w_{i}=0$ if $q_{i}\;\leq \;q_{A}$, $w_{i}=(q_{i}-q_{A})/(q_{B}-q_{A})$ if $q_{A}<q_{i}\;\leq \;q_{B}$, and $w_{i}$ = 1 if $q_{i}>q_{B}$. Choosing $q_{A}$ and $q_{B}$ for the mapping significantly reduced the optimization space and thus we can rewrite the minimization problem as:


$$\underset{q_{A},q_{B}}{\arg\min}\sum_{j=1}^{n_{0}}\ell_{-j}({\hat{\beta }}_{0-j}^{⋆})-\ell ({\hat{\beta }}_{0-j}^{⋆}),\;\text{subject}\;\text{to}\;0\;\leq \;q_{A}\;\leq \;q_{B}\;\leq \;1$$

Essentially, the weights are adaptively determined via an optimization procedure based on the data to minimize the LOOCV log-likelihood, where cancer with a similarly estimated regression coefficient (for instance, may be caused by similar etiology) will likely receive a higher weight. More details can be found in Tran *et al.* (2023).

We used the limited-memory BFGS methods (Byrd *et al.* 1995) implemented in the *optim* function in R for this optimization.

### 2.3 MGI dataset

We extracted 14 590 patients (7295 matched strata) from the MGI cohort “Freeze 4” dataset. Genotypes are obtained from array genotyping of DNA extracted from the blood sample and after TOPMed imputation and quality control (QC) filtering, the dataset encompasses over 51 million candidate variants [minor allele frequency (MAF) >0.1% and imputation accuracy Rsq >0.3] across the genome (Zawistowski *et al.* 2023). In each stratum, one cancer patient and one non-cancer control individual were matched based on gender, race, age, and ZIP code. Due to varying prevalence rates among different cancers, the sample sizes for each cancer type differ (Table 1). Regarding the extensive variant data, each variant is represented by its alternative allele dosage, a continuous variable ranging from 0 to 2, indicating the estimated number of alternative alleles at that position. The variant data is predominantly well-imputed and clusters around the values 0, 1, and 2; however, some values are non-integer, reflecting uncertainties in the imputation accuracy of those positions. In this study, we utilized only autosomal variants.

To reduce multiple testing and boost statistical power, we considered variants with an MAF observed in our dataset >5% as common variants [mean empirical imputation Rsq > 0.95 (Zawistowski *et al.* 2023)] to ensure genotypic variability for obtaining finite effect size estimates in each cancer dataset. Each of the 14 cancers was treated as a local dataset of interest, with the remaining 13 cancers serving as external datasets. For each variant, we first constructed conditional logistic regression models with genotype as the predictor and case status as the response, using only the local dataset—these are referred to as non-integrated methods. In these models, the relationship between each individual variant and cancer case–control status was detected and evaluated. Recall that in conditional logistic regression models, the likelihood is modeled at the stratum level, so including genotype as a covariate specifically means incorporating the within-stratum difference in a variant’s genotype between the case and the control. We also investigated including the matching variables of gender, race, and age in the model; however, the near-exact matching of these variables led to model instability without significantly contributing to the estimation of the variant effect.

Common variants that yielded significant nominal *P*-values in the Wald test from the non-integrated method (*P* < .05) were selected for the integration process to conserve computing power for only the most promising results. Utilizing the integration methods, we determined the weights for the external datasets and incorporated these weights into the conditional logistic regression models, using both local and external datasets. The resulting Wald test outcomes from the weighted conditional logistic regression are referred to as integrated *P*-values. We designated non-integrated *P*-values derived from a specific cancer dataset as non-integrated cancer *P*-values (e.g. non-integrated breast *P*-values) and integrated *P*-values derived from using specific cancer as the local dataset as integrated cancer *P*-values (e.g. integrated breast *P*-values).

We employed the *clogit* function from the R package *survival* to construct the conditional logistic regression models (Therneau *et al.* 2015). Within *clogit*, the weights are interpreted as case weights, specifically importance weights. These weights determine the contribution of each observation to the calculation of the approximate conditional logistic likelihood, paralleling the way information from external datasets is weighted in our integration approach. The process of using *clogit* for generating standard error estimates is validated by the fact that our integration approach and *clogit* produce identical parameter estimates when the same weights are applied. Using these standard error estimates, we derived *P*-values from the test statistic corresponding to a two-sided hypothesis test, with the null hypothesis positing that the variant’s effect size is zero.

### 2.4 FDR control

**Figure 2.**
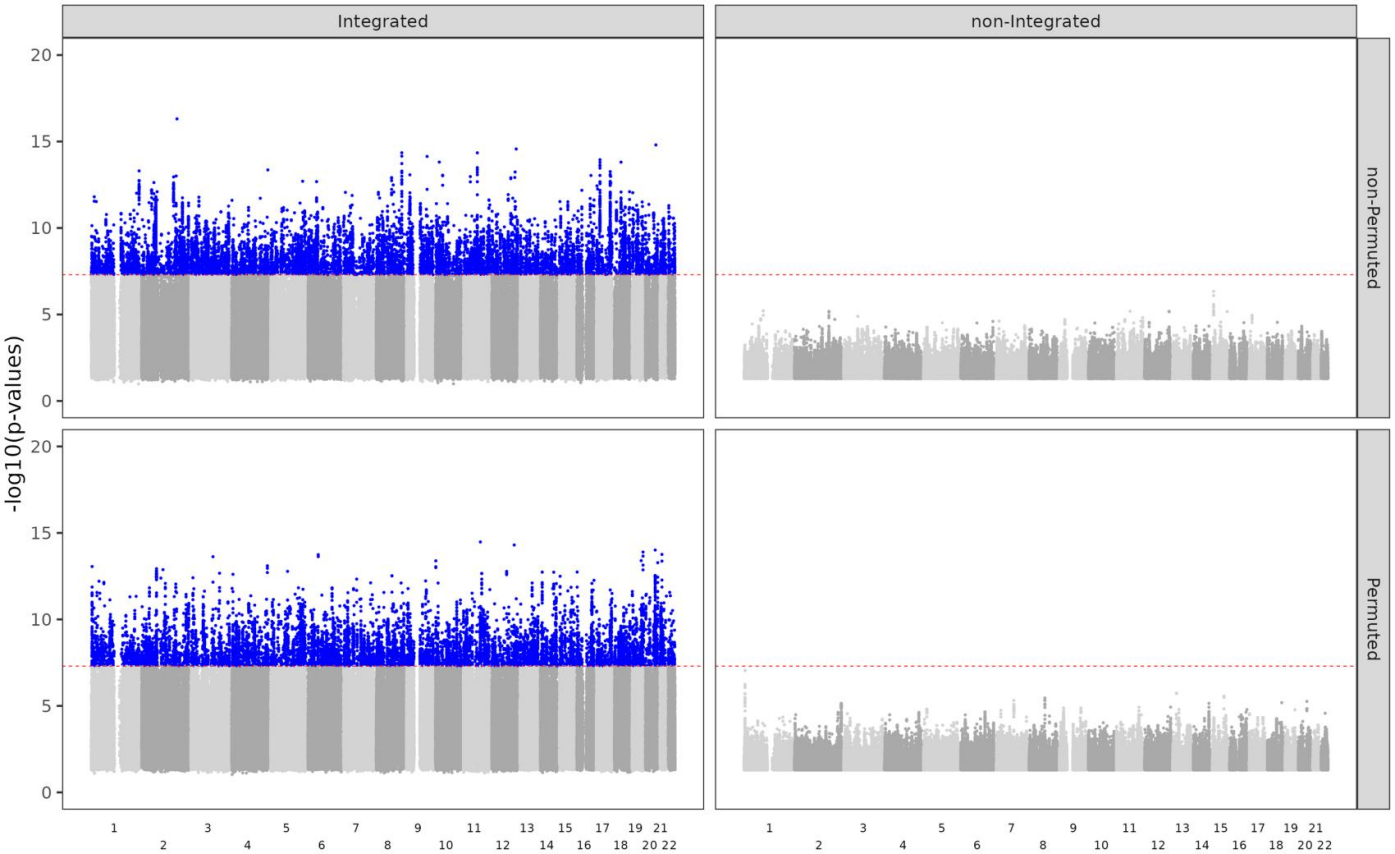
The Manhattan plot for bladder cancer displays both integrated and non-integrated *P*-values, as well as permuted and non-permuted *P*-values. The *x*-axis represents the genomic locations of the variants ordered by chromosome number, while the *y*-axis shows the -log10 *P*-values. Variants with non-integrated *P*-values ≤0.05 were selected for display. The top row presents *P*-values based on the original, non-permuted case–control status, while the bottom row shows *P*-values based on the permuted case–control status. The left column depicts post-integration *P*-values and the right column illustrates non-integrated *P*-values.

Testing millions of candidate variants necessitates a robust framework to address the multiple testing problem and control the FDR. It is important to recognize that the raw integrated *P*-values from our analysis are biased and anti-conservative (Fig. 2 and Fig. S1, available as supplementary data at *Bioinformatics Advances* online) because they were computed from conditional logistic regression models assuming fixed weights while the weights were adaptive to the data. Controlling FDR in our analysis is further complicated by genetic characteristics such as linkage disequilibrium (LD), population stratification, and admixture. All these challenges preclude the use of standard FDR-control procedures (e.g. Benjamini–Hochberg). Standard permutation tests can be used to control for FDR (Xie *et al.* 2005) but at a high computational cost. In our case, due to the large number of variants and the computationally intensive optimization procedure involved in the integration analysis, analyzing the entire dataset took months even on a computer cluster. Multiplying by many folds required by the standard permutation approach would make it extremely costly. Therefore, we adopted a single-iteration permutation method proposed in Annis *et al.* (2021).

We first randomly swapped case–control status within each stratum with 50% probability to generate a newly permuted dataset, after which we computed the corresponding permuted *P*-values. This step can provide a good estimate of the number of null findings, which helps eliminate the false discoveries due to biased *P*-values. Suppose the set of non-permuted *P*-values is *P* and the set of permuted *P*-values is $P^{⋆}$, we then sorted the sequence of non-permuted *P*-values in ascending order: $p_{1}\;\leq \;p_{2}\;\leq \;p_{3}\cdots$. Next, we defined a series of sets $\left\{P_{i}\right\}$ and $\left\{P_{i}^{⋆}\right\}$ that includes *P*-values smaller than $p_{i}$, $P_{i}=\{p:p\;\leq \;p_{i},p\in P\};P_{i}^{⋆}=\{p^{⋆}:p^{⋆}\;\leq \;p_{i},p^{⋆}\in P^{⋆}\}$. We then calculated the cardinality of each set, denoted as $m_{i}=|P_{i}|$ and $m_{i}^{⋆}=|P_{i}^{⋆}|$. The corresponding FDR estimate at the non-permuted *P*-value level $p_{i}$ is then given by ${\hat{\text{FDR}}}_{i}=\min(m_{i}^{⋆}/m_{i},1)$. Finally, we replaced ${\hat{\text{FDR}}}_{i}$ by $\min\{{\hat{\text{FDR}}}_{j},j\geq i\}$ to make them monotonically increasing.

While the non-integrated *P*-values from the conditional logistic regression are unbiased, we also computed the FDR for these non-integrated *P*-values using the same permutation method to facilitate a comparative analysis.

We note that the single-iteration permutation method may not be as robust as the standard permutation method. We conducted a smaller-scale simulation study to compare the empirical statistical power and false discover rate of the single-iteration permutation procedure with that of a 10-iteration permutation procedure. The average empirical statistical power of the two procedures are similar, while the average empirical FDR of the single-iteration procedure (0.28) is higher than that of the 10-iterations procedure (0.03). Although the FDR control is too liberal, it is still an acceptable compromise given the significant reduction in computational time. We also explored using the empirical null method to adjust the *P*-values by generating the null distribution from ten iterations. However, this approach performed poorly, likely due to the prescreening step that we adopted based on the non-integrated *P*-values, which caused the resulting z-statistics to follow a center-truncated distribution that is very difficult to model and fit. More details of these simulation study can be found in Section S3, available as supplementary data at *Bioinformatics Advances* online.

## 3 Results

We selected about 6.2 million variants with an MAF >5% as common variants out of the total 51 million variants in the MGI dataset (see Table S1, available as supplementary data at *Bioinformatics Advances* online for a detailed account of the number of common variants categorized by chromosome number). For each variant, we performed conditional logistic regression for each cancer and integrated weighted conditional logistic regression, treating each dataset as the local dataset, respectively.

Figure 2 presents the Manhattan plot for bladder cancer, displaying the selected -log10 *P*-values across the genomic locations. A comparison between the left and right columns of the figure reveals an upward shift in *P*-values post-integration across the entire genome. Additionally, comparing the top and bottom rows indicates that this effect similarly impacts both the non-permuted and the permuted datasets, justifying the use of the permuted dataset to control for FDR. Manhattan plots for all cancer types exhibited a similar trend in *P*-values. The comparison of FDR versus -log10 *P*-value of the two methods is shown in Fig. S2, available as supplementary data.

As shown in Fig. 2 and Fig. S1, available as supplementary data at *Bioinformatics Advances* online, our integrated *P*-values are highly biased, precluding their use as a standard for exploring significant associations between variants and cancers. For each set of integrated cancer *P*-values, we calculated the FDR by cancer based on the permuted *P*-values. To avoid duplicate signals in our results, we utilized LD clumping to remove the significant variants with higher LD ($R^{2}>0.5$, calculation based on 1000 Genomes Project) and closer position (<250 kb).

**Figure 3.**
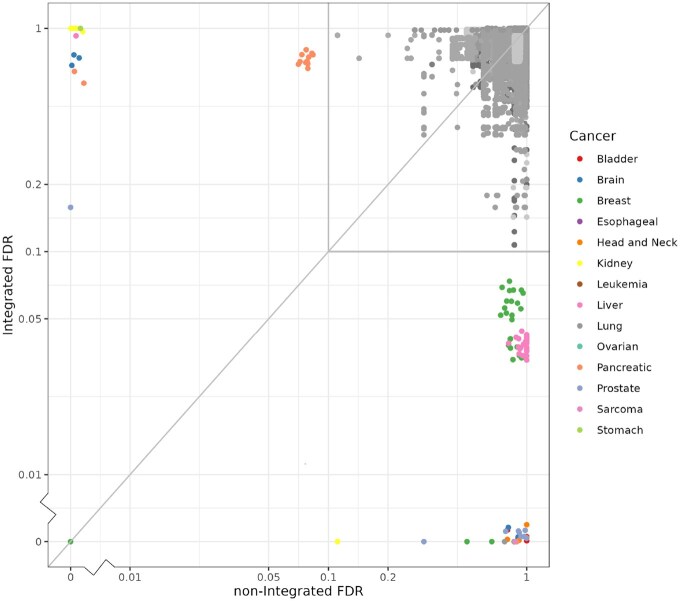
The FDR values of each variant based on integrated methods versus non-integrated methods. Colors indicate the use of different cancers as local datasets (greyed out for variants with FDR >0.1). To separate points lay exactly on each other, jittering was added to the points. The results shown in this figure are before LD clumping.

Table 2 presents the number of variants within each FDR interval after LD clumping, comparing the integrated analysis to the non-integrated analysis. Using an FDR threshold of 0.05, a total of 61 variants generated significant integrated FDR values, and 11 variants generated significant non-integrated FDR values. If we relax the FDR threshold to 0.1, 12 variants yield significant integrated FDR values and all of them used breast cancer as the local dataset. Apart from that, 6 variants, using pancreatic cancer as the local dataset, generated significant non-integrated FDR values. At the FDR threshold of 0.1, the non-integrated analysis identified fewer variants (N=17) compared with the integrated analysis (N=73), which demonstrates the potential benefit of using the integrated method. The integrated method revealed more significant variants in most cancers, especially in breast, liver, and prostate cancers. The non-integrated method identified more significant variants in kidney, pancreatic and stomach cancers. This may be due to the fact that our single-iteration permutation procedure may introduce higher variability than the standard permutation procedure and the issue was exacerbated for cancers with smaller sample sizes. Figure 3 illustrates the FDR values of the integrated analysis compared to the non-integrated analysis, highlighting that the integrated analysis tends to generate more significant FDRs.

**Table 2.** Number of variants discovered at various FDR thresholds.^a^

Cancer	Integrated analysis		Non-integrated analysis	
	*(0,0.05]*	*(0.05,0.1]*	*(0,0.05]*	*(0.05,0.1]*
Bladder	3	0	0	0
Brain	2	0	2	0
Breast	22	12	1	0
Head and neck	3	0	0	0
Kidney	1	0	3	0
Liver	19	0	1	0
Lung	1	0	0	0
Pancreatic	1	0	2	6
Prostate	7	0	1	0
Sarcoma	1	0	0	0
Stomach	0	0	1	0

a The number of significant variants in this table is lower than those in Fig. 3 as a result of LD clumping. The FDR thresholds are provided in italic values.

**Figure 4.**
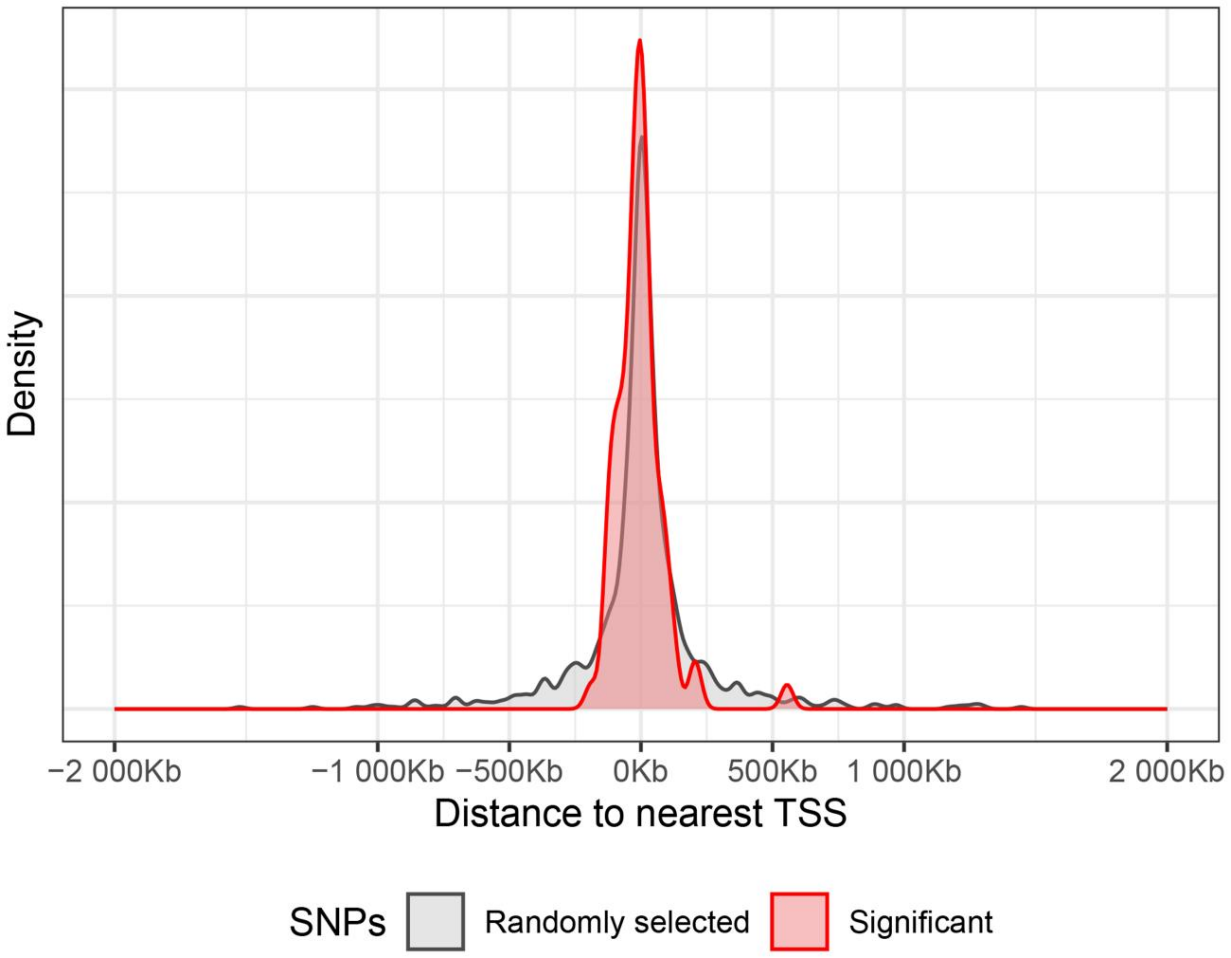
Kernel Density Estimates of the distribution of relative distances of randomly selected SNPs (N=1000) versus our identified significant SNPs (N=73) to their nearest TSS. The distance is defined as negative when the SNP is located upstream of the TSS and positive when the SNP is positioned downstream of the TSS.

**Figure 5.**
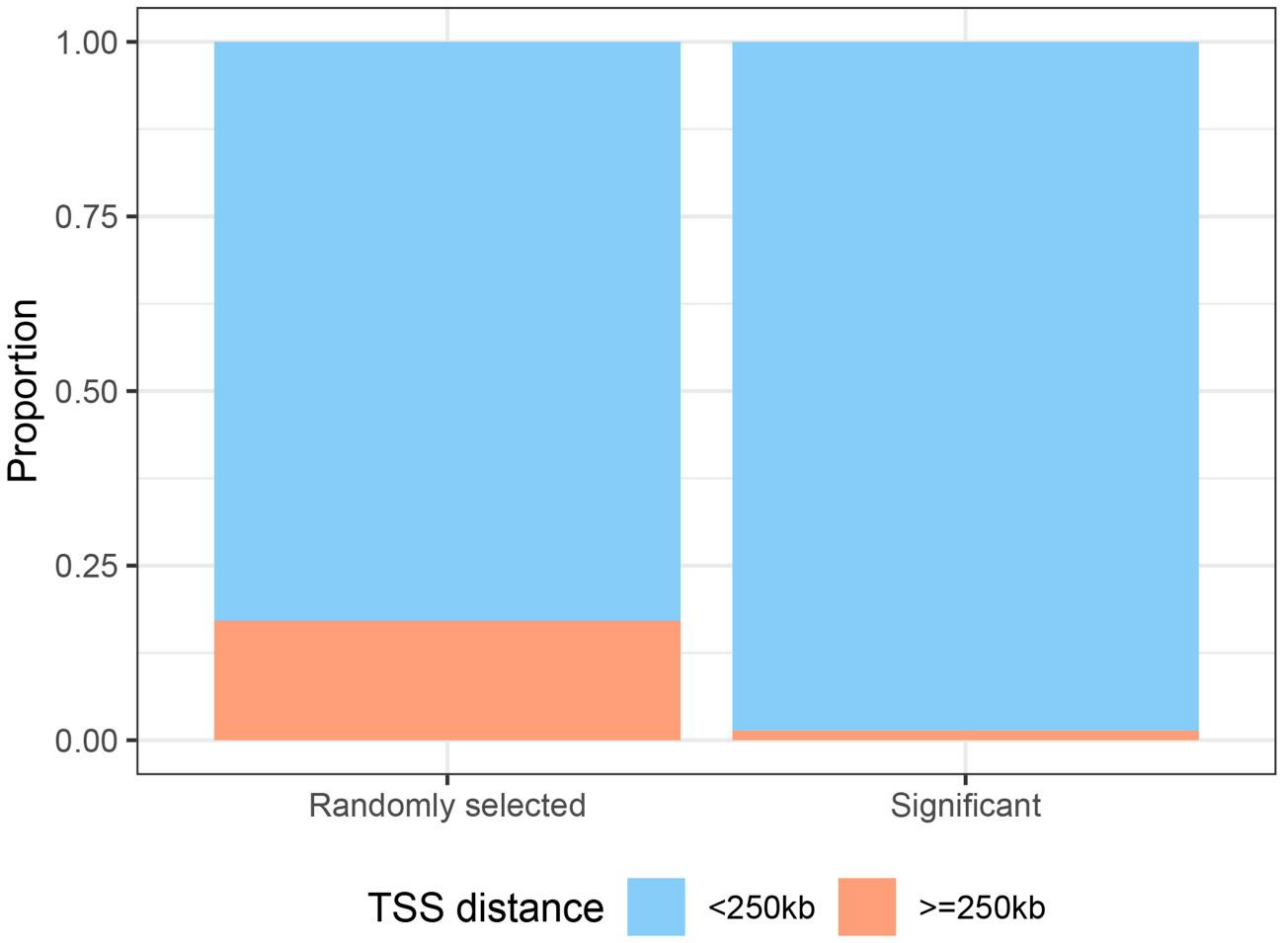
Bar plot visualizing the proportion of SNPs within ±250 kb to their nearest TSS in randomly selected SNPs versus in our identified significant SNPs.

We also examined the transcription start site (TSS) proximity of the identified single-nucleotide polymorphisms (SNPs). Figure 4 depicted the distribution of the relative distances of our identified significant SNPs (with integrated FDR  ≤  0.1) to their nearest TSS. The median distance to the nearest TSS is −2.4 kb, with 98.6% of the significant SNPs located within ±250 kb of a TSS. To assess the concentration around TSS our identified SNPs, we also estimated a null distribution using randomly selected SNPs (N=1000) from the 6 million variants in our dataset. The median relative distance of the randomly selected SNPs to their nearest TSS is 4.35 kb, with 82.9% of the randomly selected SNPs located within ±250 kb of a TSS. Therefore, the TSS of our identified SNPs is more concentrated around TSSs compared to randomly selected SNPs. We then categorized the SNPs into two groups: with absolute distance <250 kb and ≥250 kb to their nearest TSS, respectively, and compared the proportion of these two groups in Figure 5. Chi-squared test showed that there is a significant enrichment of our identified SNPs in the 250 kb window ($\chi^{2}=11.36$, P<.001).

For each identified significant variant, we also identified the neighboring gene (if available) in the single-nucleotide polymorphism database (dbSNP) and reviewed relevant literature in cancer research (see Table S2, available as supplementary data at *Bioinformatics Advances* online). Out of the 73 variants with integrated FDR  ≤ 0.1, 51 are intronic variants located within a total of 18 genes (including 3 non-coding RNAs), and the rest 22 variants are located in intergenic regions.

Furthermore, we searched our findings against the Genome-Wide Association Studies (GWAS) catalog (https://www.ebi.ac.uk/gwas/) (Cerezo *et al.* 2025), UKBiobank and MGI PheWebs (https://pheweb.org/) (Gagliano Taliun *et al.* 2020). Of the 73 identified variants, 10 had been previously reported to have significant associations ($P<5\times 10^{-8}$) with known traits. All of them were related to breast cancer. Eight of the breast cancer-associated variants are intronic variants in the *TOX3* gene, one is a intronic variant in the *CASC16* gene, while the remaining one is located in the intergenic region between *TOX3* and *CASC16*. Additionally, literature search of the 18 associated genes revealed that 9 of them are linked to cancer.

For instance, rs112149573, which exhibited an interesting yet non-significant (at a GWAS threshold of $5\times 10^{-8}$) association with breast cancer in the non-integrated conditional logistic regression (*P* = $2.48\times 10^{-7}$, FDR = 0.5), was found to be significant at FDR < 0.04 in our integrated analysis. This SNP is an intronic variant and genic upstream transcript variant of the TOX High Mobility Group Box Family Member 3 (*TOX3*) gene. *TOX3* is well-documented for its association with breast cancer through multiple SNPs (Zhang and Long 2015). In this case, the integrated FDR corroborates the raw *P*-value, and the result is consistent with prior findings. More detailed information (*P*-value, SNP location, FDR, etc.) can be found in Table S2, available as supplementary data at *Bioinformatics Advances* online.

Additionally, the integration method revealed enhanced associations between some variants and cancers that were less pronounced in the non-integrated analysis, potentially highlighting significant findings. For example, several intronic variants within the DEP domain containing 1B (*DEPDC1B*), such as rs10939856 and rs1379116, displayed *P*-values around 0.03 in the non-integrated conditional logistic regression analyses with the liver cancer dataset. However, incorporating additional cancer datasets improved the observed associations between these variants and liver cancer. This finding is consistent with prior research demonstrating upregulation of *DEPDC1B* expression in hepatocellular carcinoma (Shen *et al.* 2022).

## 4 Discussion

In this study, we developed a novel asymmetric data integration method for matched case–control data analysis. This approach allowed us to integrate datasets from different cancers and fit conditional logistic regression models with data-adaptive weights. A comparison of the results from the integration method with those from conventional non-integrated conditional logistic regression analysis revealed an increased number of significant findings of potential cancer risk-associated common variants and related genes. Many of these genes have been previously identified in the cancer literature, thereby supporting our findings.

As we know that population stratification might potentially confound genetic studies like this, we did one-to-one matching on race alongside age, sex, and ZIP code in our case–control design, aimed at controlling for the potential confounding effect of population stratification. We did not adjust for additional population factors beyond this due to computational considerations. While there could still exist residual population factors, their effects on FDR could be further controlled by our permutation procedure as the permuted data has the same genotypes and population factors. We will investigate more efficient ways to control for population stratification in future studies.

We recognize that the single-iteration permutation method used for FDR control was less robust than ideal, as indicated in our simulation study. Nevertheless, this approach was adopted for computational benefits. In future work, we aim to develop or identify a more effective FDR control method that better balances computational efficiency with robustness in FDR control.

We have also tried to apply our methods on pathogenic variants which have lower MAFs with tests based on burden scores (see Section S4, available as supplementary data at *Bioinformatics Advances* online). However, the result is not as prominent as those for common variants, likely due to low power. Future research will be directed toward refining the application of this method to pathogenic variants to improve power.

## Supplementary Material

vbaf253_Supplementary_Data

## Data Availability

The MGI data underlying this article cannot be shared publicly due to patient confidentiality. However, the MGI data are available from the Michigan Genomics Initiative at https://aidhi.umich.edu/mgi-community for researchers who meet the criteria for confidential data access.
