# Supplementary material for: Asymmetric integration of various cancer datasets for identifying risk-associated variants and genes
Source: Bioinform Adv. 2025 Oct 14;5(1):vbaf253. doi: 10.1093/bioadv/vbaf253 (PMC12576323; doi:10.1093/bioadv/vbaf253)
Supplement: vbaf253_Supplementary_Data [file vbaf253_supplementary_data.pdf]

Supplementary Materials: Asymmetric Integration of Various Cancer Datasets for Identifying Risk-Associated Variants and Genes

Ruixuan Wang[1], Lam Tran[1], Benjamin Brennan[1], Lars G. Fritsche[1,2], Kevin He[1], J. Chad Brenner[3], and Hui Jiang[1,2,*]

[1]Department of Biostatistics, University of Michigan, Ann Arbor, MI
[2]Center for Statistical Genetic, University of Michigan, Ann Arbor, MI
[3]Department of Otolaryngology-Head and Neck Surgery, University of Michigan, Ann Arbor, MI
[*]Corresponding author: Hui Jiang, jianghui@umich.edu

S.1 Supplementary tables

See Table S.1 for the number of common variants, categorized by chromosome number.

Table S.1: Variants with MAF $>$ 5% by chromosome

Chr	# Variants	Chr	# Variants
1	477781	12	300525
2	517001	13	233503
3	444643	14	200769
4	457178	15	174029
5	402877	16	185914
7	366503	17	161442
6	420464	18	177571
8	344008	19	137022
9	268634	20	137584
10	322506	21	86595
11	316165	22	80128

See Table S.2 for detailed information on variants with significant FDR[1] values.

[1]To lean on the conservative side, if a variant has an estimated $\widehat{FDR}$ of 0, we denote its FDR estimate as being less than the smallest non-zero FDR estimates in that cancer, but still includes it in the $(0, 0.05]$ statistical bin.

Table S.2: Significant variants (after LD clumping) with FDR ≤ 0.05 in integrated analysis ($N = 61$)

Cancer	SNP ID	Chromosome	Position	Non-Integrated		Integrated		Gene
				p-value	*FDR*[1]	*p-value*	*FDR*[1]	
Bladder	rs274856	20	53413591	1.76E-04	1.00	1.58E-15	<0.2	TSHZ2
Bladder	rs4616136	12	124968217	3.37E-02	1.00	2.69E-15	<0.2	DHX37
Bladder	rs4538161	2	180864301	3.77E-02	1.00	4.89E-17	<0.2	SCHLAP1
Brain	rs13251533	8	66254981	2.26E-02	0.93	1.49E-16	<0.33	LOC102724687
Brain	rs13253568	8	66254982	2.26E-02	0.93	1.37E-16	<0.33	LOC102724687
Breast	rs4784226	16	52549231	1.97E-07	<0.5	7.03E-16	<0.04	TOX3
Breast	rs112149573	16	52547333	2.48E-07	0.50	1.23E-15	<0.04	TOX3
Breast	rs4784227	16	52565276	3.27E-07	0.67	8.75E-15	<0.04	CASC16
Breast	rs10109041	8	130550773	3.04E-04	0.94	6.22E-15	<0.04	LOC105375758
Breast	rs3112578	16	52551528	1.76E-06	1.00	2.54E-15	<0.04	/
Breast	rs45465998	16	52549498	2.60E-06	1.00	2.10E-15	<0.04	TOX3
Breast	rs7500427	16	52511365	4.98E-06	1.00	1.88E-15	<0.04	TOX3
Breast	rs1362548	16	52530039	5.76E-06	1.00	5.57E-15	<0.04	TOX3
Breast	rs111748199	1	9274836	6.39E-06	1.00	5.65E-15	<0.04	/
Breast	rs9936081	16	52515734	6.80E-06	1.00	4.25E-15	<0.04	TOX3
Breast	rs11935541	4	13283070	5.69E-05	1.00	1.84E-15	<0.04	/
Breast	rs61925909	12	25314598	7.41E-05	1.00	6.33E-15	<0.04	/
Breast	rs648399	1	10496428	1.32E-04	1.00	1.22E-16	<0.04	PEX14
Breast	rs11032549	11	34206421	1.61E-04	1.00	3.94E-15	<0.04	ABTB2
Breast	rs2975526	8	130570345	2.08E-04	1.00	5.09E-15	<0.04	/
Breast	rs622623	1	10495200	2.18E-04	1.00	5.52E-16	<0.04	PEX14
Breast	rs10185517	2	680661	4.69E-04	0.91	1.37E-14	0.04	TMEM18-DT
Breast	rs2975534	8	130560769	3.59E-04	0.92	1.96E-14	0.04	/
Breast	rs2979008	8	130571187	3.27E-04	0.93	1.20E-14	0.04	/
Breast	rs2959317	8	130567288	3.23E-04	0.93	1.94E-14	0.04	/
Breast	rs2975528	8	130567902	3.12E-04	0.94	1.49E-14	0.04	/
Breast	rs12459882	19	43064117	2.76E-05	1.00	3.14E-14	0.04	PSG2
Breast	rs9866146	3	193716588	6.97E-05	1.00	3.42E-14	0.04	/
Head and Neck	rs6414490	3	181602608	7.86E-04	1.00	8.80E-16	<0.25	SOX2-OT
Head and Neck	rs13253568	8	66254982	4.14E-03	1.00	5.01E-17	<0.25	LOC102724687
Head and Neck	rs13251533	8	66254981	4.15E-03	1.00	1.19E-16	<0.25	LOC102724687
Kidney	rs6946141	7	24886215	2.36E-06	0.11	3.20E-16	<0.4	OSBPL3
Liver	rs7719611	5	60692720	5.42E-03	0.91	3.39E-15	0.04	DEPDC1B
Liver	rs7704338	5	60690328	5.42E-03	0.91	1.71E-15	0.04	DEPDC1B
Liver	rs11748965	5	60681591	5.49E-03	0.91	1.58E-15	0.04	DEPDC1B
Liver	rs11742421	5	60681590	5.52E-03	0.91	1.64E-15	0.04	DEPDC1B
Liver	rs78304903	5	60569539	1.06E-02	0.96	1.60E-14	0.04	/
Liver	rs17178530	17	71239971	1.43E-02	0.98	3.70E-14	0.04	/
Liver	rs11955398	5	60685258	2.07E-02	1.00	4.52E-15	0.04	DEPDC1B
Liver	rs60148983	5	60559205	2.39E-02	1.00	4.93E-15	0.04	/
Liver	rs55768631	5	60581364	2.40E-02	1.00	7.05E-15	0.04	/
Liver	rs58799235	5	60559276	2.40E-02	1.00	6.72E-15	0.04	/
Liver	rs28489600	18	36639606	2.69E-02	1.00	4.31E-14	0.04	FHOD3
Liver	rs17387940	5	60671889	2.83E-02	1.00	1.01E-14	0.04	DEPDC1B
Liver	rs59155887	5	60665716	2.83E-02	1.00	1.37E-14	0.04	DEPDC1B
Liver	rs1379116	5	60646573	2.85E-02	1.00	2.41E-14	0.04	DEPDC1B
Liver	rs11741754	5	60681231	2.89E-02	1.00	1.32E-14	0.04	DEPDC1B
Liver	rs10939856	5	60634469	3.24E-02	1.00	1.84E-14	0.04	DEPDC1B
Liver	rs62373027	5	60620516	3.26E-02	1.00	1.74E-14	0.04	DEPDC1B
Liver	rs112995339	5	9947107	3.54E-02	1.00	5.28E-15	0.04	LOC107986405
Liver	rs4585378	4	188328694	3.92E-02	1.00	3.37E-14	0.04	/
Lung	rs13158665	5	60773230	9.30E-04	0.78	2.20E-18	<1	ELOVL7
Pancreatic	rs13158665	5	60773230	4.38E-03	0.90	9.41E-18	<1	ELOVL7
Prostate	rs4653250	1	33596298	2.52E-06	0.30	5.75E-16	<0.11	CSMD2
Prostate	rs7653134	3	169876322	9.37E-03	0.98	6.34E-15	<0.11	/
Prostate	rs669701	1	10484668	1.30E-02	0.97	1.89E-15	<0.11	PEX14
Prostate	rs620405	1	10494737	1.04E-02	0.98	1.55E-16	<0.11	PEX14
Prostate	rs34647980	1	10483760	1.35E-02	0.98	2.27E-15	<0.11	PEX14
Prostate	rs662064	1	10497194	1.08E-02	0.98	2.79E-15	<0.11	PEX14
Prostate	rs10214936	7	138428403	4.33E-03	0.98	5.77E-16	<0.11	/
Sarcoma	rs4653250	1	33596298	7.53E-04	0.92	1.70E-16	<1	CSMD2

S.2 Supplementary figures

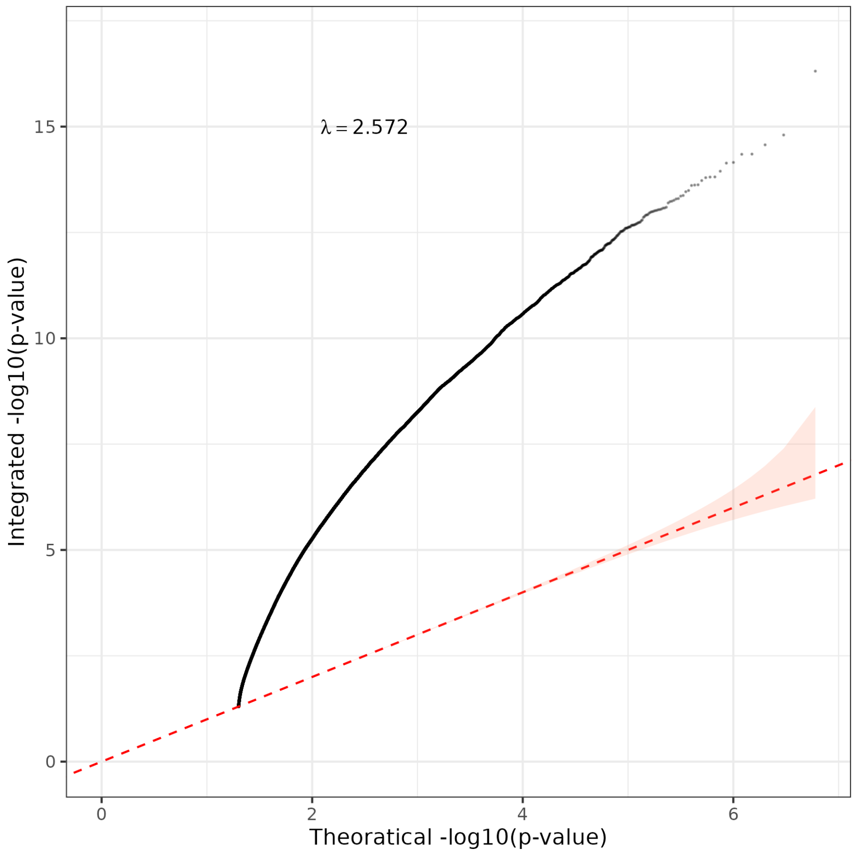

Figure S.1: A QQ-plot for the distribution of the integrated p-values with bladder cancer as the primary dataset against expected uniform p-values. The x-axis represents the expected -log10 p-values, while the y-axis represents the integrated -log10 p-values. SNPs with integrated p-value < 0.05 are selected. An significant bias in integrated p-value is observed in that all points are way above the red dash diagonal line.

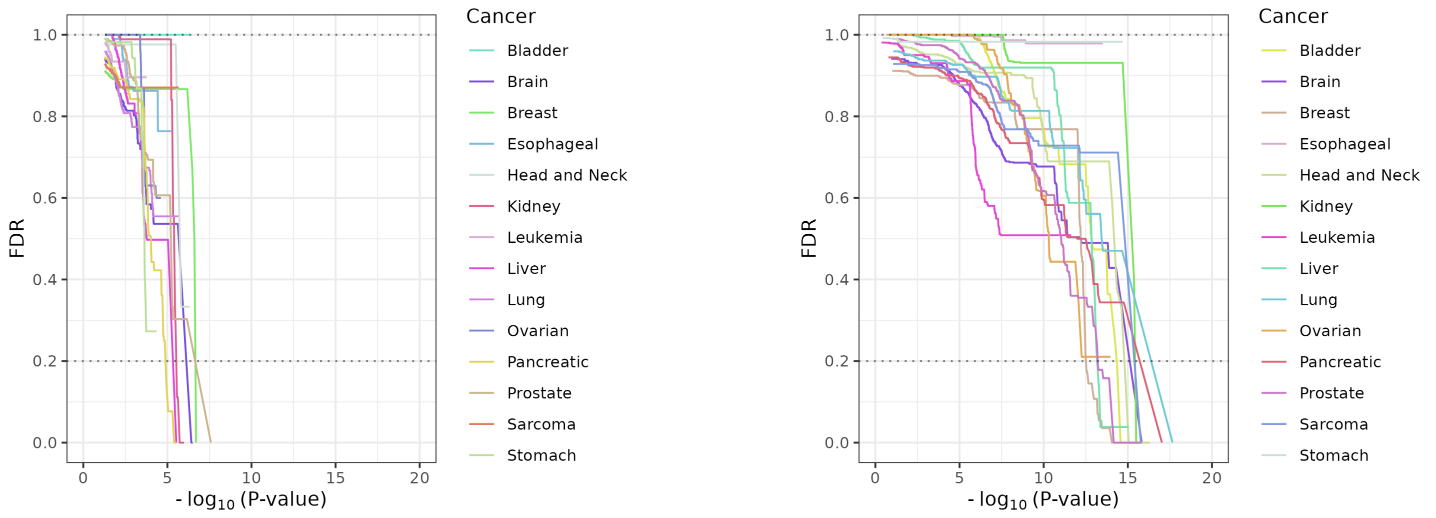

(a) Non-integrated analysis

(b) Integrated analysis

Figure S.2: Estimated FDR (y-axis) vs. -log10 p-value threshold (x-axis) for each cancer for non-integrated (left) and integrated analyses (right).

S.3 Simulation study on single-iteration permutation method

We conducted a simulation study using our available genotype data. From the full set of 6.2 million SNPs, we selected 6,206 by taking every 1,000th SNP based on chromosome number and position. Among these, 20 SNPs were randomly designated as true signals, with the remaining treated as null. For the true signals, we assigned significance as follows: 5 SNPs in 1 random cancer, 5 in 2 random cancers, 5 in 3 random cancers, and the remaining 5 in 4 random cancers.

True effect sizes (betas from conditional logistic regression) were sampled from the distribution of our significant variants. For each of the 20 true signals, we randomly selected effect sizes for the cancers in which they were assumed to be significant. To amplify the signal, we increased the betas by a factor of 20. Given that SNPs often influence multiple cancers in the same direction, we ensured the assigned betas had consistent signs across cancers.

Finally, we simulated case-control status using the following formula. For each stratum j, the logit of the case probability was calculated as the sum of β times the genotype difference $(X_{1ij}–X_{2ij})$ across the 20 true signals:

$$log(\frac{P(Case_j = 1)}{P(Control_j = 1)}) = \sum_{i=1}^{20} \beta_i * (X_{1ij}–X_{2ij})$$

The original beta values were estimated from single-variant conditional logistic regression, but the case-control status was simulated using a multi-variant conditional logistic regression model.

We then applied the methodology outlined in Figure 1 to the selected 6,206 SNPs and computed the false discovery rate (FDR) for each. Using an FDR threshold of < 0.1, we evaluated both statistical power and FDR. To address computational constraints, we conducted 10 permutations in total and compared the results from a single-iteration permutation with those from a ten-iteration permutation. The average FDR for the single-iteration permutation was 0.283, with an average statistical power of 0.845. In contrast, the ten-iteration permutation yielded a substantially lower FDR of 0.035, with a slightly reduced power of 0.800. While the FDR control from the single-iteration permutation was suboptimal, it reflects a trade-off between computational efficiency and statistical accuracy.

S.3.1 Empirical null method using simulation study

Empirical null method is a commonly used statistical approach to estimate the false discovery rate. We obtained the null distribution of the Wald test z-statistics ($z = \frac{\hat{\beta}}{SE(\hat{\beta})}$) from the weighted conditional logistic regressions in our permuted dataset of the simulation study. As shown in Figure S.3, the resulting distribution of the Wald test z-statistics exhibits a nearly symmetric, bimodal shape. This pattern arises because our filtering step removes SNPs

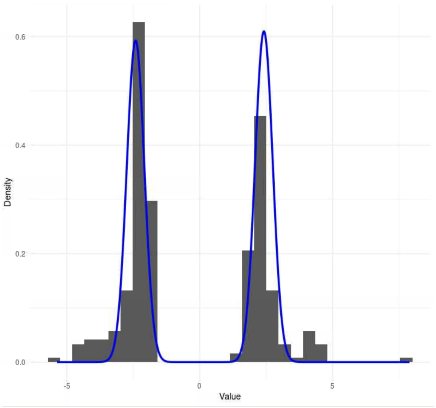

(a) Gaussian Mixed Model

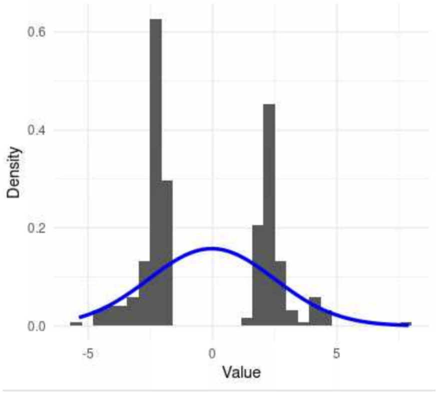

(b) Gaussian Model

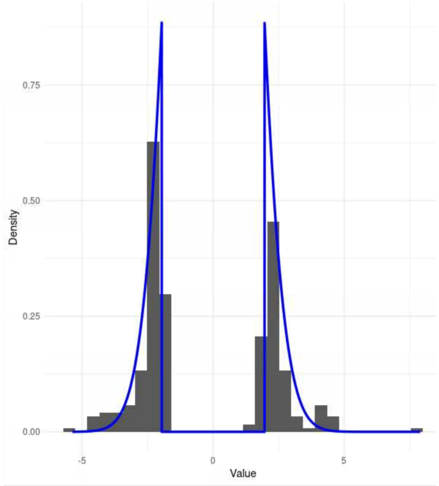

(c) Truncated Gaussian Model (threshold = 1.96)

Figure S.3: Histogram of z-statistics of simulation study of bladder cancer with the fitted parametric distribution. (a) Gaussian mixed model, (b) Gaussian model, (c) truncated Gaussian model(threshold = 1.96).

with smaller absolute Wald statistics, which would otherwise contribute to the center of the distribution.While this filtering step was necessary to improve computational efficiency, it makes the traditional empirical null method with normal distribution hypothesis less appropriate for our analysis.

We applied three models to the null distribution: a Gaussian mixture model (Figure S.3(a)), a standard Gaussian model (Figure S.3(b)), and a truncated Gaussian model with a threshold of 1.96 (Figure S.3(c)). For each model, we computed p-values based on the corresponding empirical distribution and adjusted them using the Benjamini–Hochberg (BH) procedure to control the false discovery rate (FDR). As shown in Figure S.3, none of the models provided a good fit to the observed null distribution.

We then evaluated the empirical null method in terms of statistical power and FDR. The Gaussian mixture model achieved a power of 0.6 but had a high FDR of 0.7. The Gaussian model showed a low power of 0.3 and an FDR of 0, likely due to the truncation of central values in the distribution, which caused the variance to be overestimated. The truncated Gaussian model yielded a power of 0.45 and an FDR of 0.6. Overall, none of the models demonstrated satisfactory performance in terms of both statistical power and FDR control. These results suggest that the empirical null approach is not a reliable alternative to the single-iteration permutation method in this setting.

S.4 Applying integration methods on pathogenic variants

S.4.1 Methods

For pathogenic variants with low MAF, we implemented specific integration rules to ensure that at least one genotype was represented in each cancer type. We integrated the number of mutations within these gene regions based on the location of the variants. Given the high number of relatively rare variants, we selected only known pathogenic mutations to aggregate as pathogenic variants at the gene level. ClinVar, a freely available public archive, provides human genetic variants and their relationships to diseases and other conditions [1]. As of the 2023-05 version, ClinVar reported a total of 17,911 human genes. Using the start and end positions of genes, we filtered out pathogenic variants present in our dataset. For each specific gene, we calculated a gene score, representing the number of ClinVar-reported pathogenic variants of that gene in each patient, with possible values of 0 or 1. While theoretically, a patient could have multiple pathogenic mutations at different positions within a gene, our dataset contained a maximum of one pathogenic variant per patient. Despite combining all pathogenic variants, some genes had 0 pathogenic variants in the local dataset, resulting in a zero design matrix.

Following the approach applied to common variants, we constructed both non-integrated and integrated conditional logistic regression models for each cancer, treating each as a local dataset. However, due to the presence of zero design matrices, some conditional logistic regression models did not converge when certain cancers were used as local datasets. To address this issue, we assigned a weight of zero to external datasets that lacked non-integrated p-values to prevent computational errors. Consequently, not all integration models utilized all 13 external datasets.

S.4.2 Results

Among the 17,911 human genes listed in ClinVar, we identified 1,881 genes with reported pathogenic variants having non-zero allele counts in our dataset. However, only 1,252 of these genes generated at least one cancer-integrated p-value. Notably, 29 genes produced cancer-integrated p-values for all 14 cancers. Due to variations in sample sizes, the number of cancer-integrated p-values per gene varies, as detailed in Table S.3. Cancers with larger sample sizes tend to yield a greater number of genes with integrated p-values.

Table S.3: # genes in integrated pathogenic variant analysis.

Cancer Type	# Genes	Cancer Type	# Genes
Bladder	402	Liver	246
Brain	282	Lung	296
Breast	753	Ovarian	176
Esophageal	171	Pancreatic	201
Head and neck	441	Prostate	576
Kidney	456	Sarcoma	418
Leukemia	90	Stomach	95

The Manhattan plots and FDR plots for pathogenic variants exhibited a trend similar to that observed for common variants, thereby reinforcing the validity of our results. However, no genome-wide significant association was identified. Given the low MAF of pathogenic variants, calculating conditional logistic regression for cancer datasets with a gene score of 0 in both case and control groups is not meaningful. Furthermore, an excessive number of zeroes in the design matrix limits the effectiveness of the integration process, resulting in external dataset weights of only 0 or 1, rather than values between 0 and 1.

For instance, consider the BRCA1 gene (Fig. S.4), a tumor suppressor gene located on chromosome 17, which is known to be associated with an increased risk of breast, ovarian, prostate, and other cancers [2]. As expected, the gene scores for BRCA1 pathogenic variants differ noticeably in breast cancer between case and control groups. Even when combining all pathogenic variants, the highest prevalence of BRCA1 pathogenic variants in each cancer dataset is below 1.5%, leading to non-significant results after controlling for multiple tests. The non-integrated conditional logistic regression yielded a p-value of 0.033, while the integrated methods produced consistent p-values of 0.005 for breast, liver, and lung cancers as the local dataset. This consistency is attributed to the low MAF, which rendered the original conditional logistic regression unreliable and resulted in uniform weights of 1 for the three cancers, irrespective of which dataset was used as the local dataset.

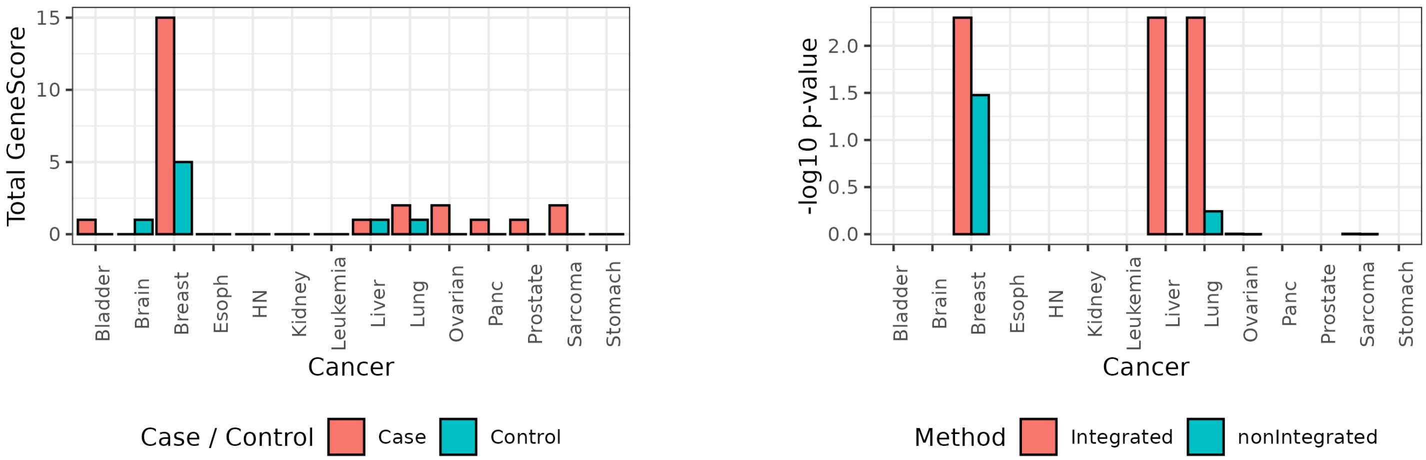

Figure S.4: Total gene score and -log10 p-values of each cancer for BRCA1 in pathogenic variant analysis. Top: total gene scores of BRCA1 in each cancer dataset; bottom: -log10 p-values of BRCA1 in each cancer dataset.

References

[1] Melissa J Landrum et al. "ClinVar: improvements to accessing data". In: *Nucleic acids research* 48.D1 (2020), pp. D835–D844.

[2] William D Foulkes and Andrew Y Shuen. "In brief: BRCA1 and BRCA2". In: *The Journal of pathology* 230.4 (2013), pp. 347–349.
